# Distribution Systems of Insecticide-Treated Bed Nets for Malaria Control in Rural Burkina Faso: Cluster-Randomized Controlled Trial

**DOI:** 10.1371/journal.pone.0003182

**Published:** 2008-09-11

**Authors:** Olaf Müller, Manuela De Allegri, Heiko Becher, Justin Tiendrebogo, Claudia Beiersmann, Maurice Ye, Bocar Kouyate, Ali Sie, Albrecht Jahn

**Affiliations:** 1 Department of Tropical Hygiene and Public Health, Ruprecht-Karls-University Heidelberg, Heidelberg, Germany; 2 Centre de Recherche en Santé de Nouna, Nouna, Burkina Faso; 3 Centre National de Recherche et de la Formation au Paludisme, Ouagadougou, Burkina Faso; Walter and Eliza Hall Institute of Medical Research, Australia

## Abstract

**Background:**

Insecticide-impregnated bed nets (ITNs) have been shown to be a highly effective tool against malaria in the endemic regions of sub-Saharan Africa (SSA). There are however different opinions about the role of ITN social marketing and ITN free distribution in the roll-out of ITN programmes. The objective of this study was to evaluate the effects of free ITN distribution through antenatal care services in addition to an ITN social marketing programme in an area typical for rural SSA.

**Methods:**

A cluster-randomised controlled ITN trial took place in the whole Kossi Province in north-western Burkina Faso, an area highly endemic for malaria. Twelve clusters were assigned to long-term ITN *(Serena brand)* social marketing plus free ITN *(Serena brand)* distribution to all pregnant women attending governmental antenatal care services (group A), and 13 clusters to ITN social marketing only (group B). The intervention took place during the rainy season of 2006 and thereafter. The trial was evaluated through a representative household survey at baseline and after one year. *Serena* ITN household ownership was the primary outcome measure.

**Findings:**

A total of 1052 households were visited at baseline in February 2006 and 1050 at follow-up in February 2007. Overall *Serena* ITN household ownership increased from 16% to 28% over the study period, with a significantly higher increase in group A (13% to 35%) than in group B (18% to 23%) (p<0.001).

**Interpretation:**

The free distribution of ITNs to pregnant women through governmental antenatal care services in addition to ITN social marketing substantially improved ITN household ownership in rural Burkina Faso.

**Trial registration:**

Controlled-Trials.com ISRCTN07985309

## Introduction

Malaria remains the most important parasitic disease in the world [1]. Every year, there are some 5 billion clinical episodes resembling malaria, some 600 million clinical malaria cases and about 1 million malaria deaths [2]. Most malaria deaths occur in young children of rural sub-Saharan African (SSA) areas with little access to health services 3–6. Early treatment with effective antimalarial drugs and sustainable vector control methods remain the main tools for malaria control [1].

Insecticide-treated bed nets (ITNs) as a new tool in malaria control have received considerable interest over the last two decades. A number of large-scale randomised controlled trials, in which children have partly been followed-up for extended periods of time, have consistently demonstrated a sustainable efficacy of ITNs in reducing malaria morbidity and mortality over a broad range of malaria transmission intensities in SSA [7]–[12]. Moreover, technical progress has now enabled the development of reliable long-lasting insecticide-treatment, both for the production of long-lasting insecticidal nets (LLIN) [13]–[17] and as impregnation or re-impregnation with an insecticide formulation [18].

ITNs were already employed on a large-scale since the 1980s in a number of malaria endemic areas of Asia, where they have contributed to major successes in malaria control [19]. Today, the need for a large scale utilisation of ITNs in SSA is well accepted in the international scientific community [20]–[24]. A high coverage with ITNs would also lead to a mass effect on the mosquito populations similar to what can be achieved through systematic insecticide spraying programs [25]–[27]. However, due to major problems with infrastructure, public service organisation, funds and leadership, progress in the implementation of ITN programs in SSA remains slow [28], [29].

Two approaches for scaling up ITN coverage in SSA are being discussed; one considers ITNs as a public good that should be provided free of charge [22]. The other is to strengthen commercial markets while providing subsidies for the groups most at risk, such as children and pregnant women [23]. Those in favour of free ITN distribution support their argumentation by the evidence from a number of SSA projects and programmes regarding the feasibility of such an approach, the proof of a significant community effect in most areas with high ITN coverage, the reality of a high proportion of SSA populations being unable to pay for such an intervention, and the hope that rich countries would sustain their financial commitment for malaria control in SSA [22]. On the other side, those in favour of strengthening commercial markets support their argumentation by the success of a large ITN social marketing programme in rural Tanzania [30]–[32], the important role of market involvement in the success of ITN programmes in Asia, the assumption that free ITN provision would destroy local commercial markets, and the uncertainty of a continuous availability of external funds for ITN programmes [23]. There is, however, considerable agreement in both groups regarding the need for major donor assistance for whatever approach [22], [23], [33]. As an alternative strategy, it has been proposed to combine free ITN distribution through antenatal care services with social marketing [34].

This study provides data from a randomized controlled community intervention on the effects of subsidised ITN distribution through a social marketing system with or without free ITN distribution through governmental health services on ITN ownership and use in Burkina Faso.

## Methods

The protocol for this trial and supporting CONSORT checklist are available as supporting information; see Checklist S1 and Protocol S1.

### Study area

The study took place in Nouna Health District (NHD), which is equivalent to the Kossi province, in rural north-western Burkina Faso. The study was implemented through the *Centre de Recherche en Santé de Nouna* (CRSN), which is based in Nouna town, the capital of NHD. By the end of 2006, NHD had a population of 304 150 living in some 300 villages. The Nouna area is a dry orchard savannah, populated mainly by subsistence farmers of different ethnic groups. Malaria is holoendemic but highly seasonal, and the annual Entomological Inoculation Rate (EIR) varies between 100 and 1000 in the different villages [35], [36]. The rainy season usually lasts from June until October.

**Figure 1:**
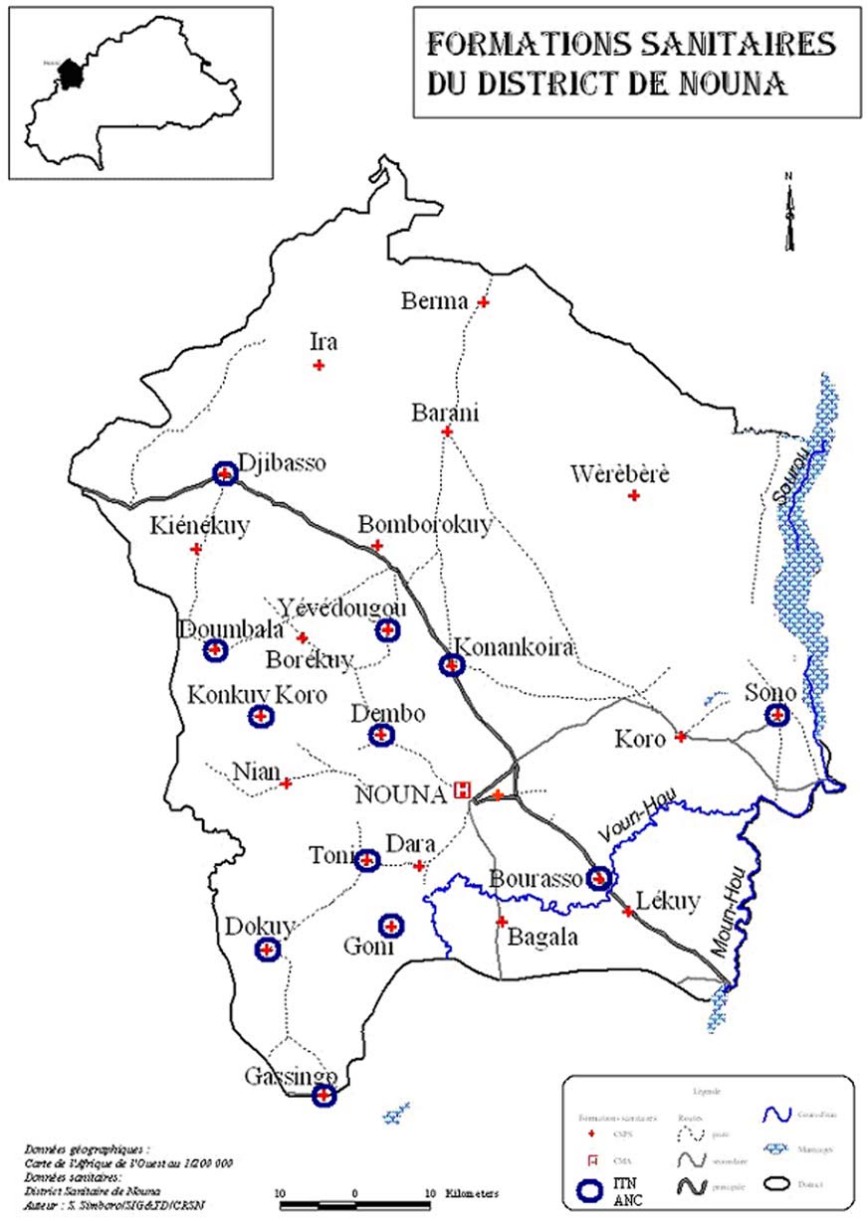
Map of the study area in the Nouna Health District. The health-centre defined intervention areas are marked with a red cross (intervention area A = circles; intervention area B = no circles).

Formal health services are limited to 24 village-based health centres and a health centre and hospital in Nouna town (Figure 1). The village-based health centres are usually staffed by two nurses and are responsible for a catchment area of between 5 and 23 villages [29]. Malaria control is thus based on home treatment with locally available drugs, which is mainly chloroquine until today, despite a recent change of the national guidelines to artemisinin-based combination therapy (ACT) as first-line drugs [6], [29]. In the recent past, roughly one quarter of young children were usually protected with untreated bed nets in NHD during rainy seasons [35], [37]. Due to a preceding efficacy trial, ITNs are already introduced to the 41 villages of the CRSN research area surrounding Nouna town [12], [38]. Moreover, the NGO Population Services International (PSI) had sold ITNs since 2002 nearly exclusively in Nouna town without subsidy for 4250 CFA (6.5 Euro; 1 Euro = 650 CFA).

### Study design

The study was designed as a community-based cluster-randomised controlled trial. The district was divided into 24 clusters on the basis of the catchment area of peripheral health centres and randomised to intervention A (social marketing of ITNs to the general population plus free distribution of ITNs to pregnant women attending antenatal care services; 12 clusters) and intervention B (only social marketing of ITNs to the general population; 12 clusters). Moreover, one additional cluster comprising the town of Nouna and neighbouring villages was pre-assigned to receive intervention B.

### Study interventions

With funds from the National Malaria Control Programme and starting in August 2006, PSI sold highly subsidised ITNs through village-based shops in the whole of NHD. The branded product is called *Serena*, which is a deltamethrin-impregnated white family-size long-lasting ITN (PermaNet, Vestergaard Frandsen, Denmark). Sales were supported by an extended social marketing campaign, including radio and television messages, poster exhibition, and community-based information, education, and communication (IEC) activities. At the beginning of the campaign, PSI also conducted promotion visits to potential sellers. The aim was to create demand for the branded product by convincing the public of its utility and informing shop-keepers of its availability in the town of Nouna. PSI in fact, sold ITNs exclusively to one wholesaler in Nouna and directed the single shop-keepers to him for further purchases. Over the period 8/2006 until 2/2007, a total of 15.000 ITNs (estimated need for the intervention period) were made available through PSI for subsidized sale in NHD. The white family size *Serena* ITN was sold for 1500 CFA ( = 2.3 Euro) to the population.

Also since August 2006 and in parallel to the ITN distribution through PSI, free distribution of the *Serena* ITN took place through health centre-based governmental antenatal care services in the areas of intervention A. The nurses of the respective health centre received their ITNs regularly from the District Health Team and provided them free of charge to all pregnant women attending antenatal care services and who lived in the defined catchment area of these health centres. A total of about 3 500 ITNs (estimated need for the intervention period, based on population size and birth rates) were distributed through health centres over the period 8/2006 until 2/2007.

### Study evaluation

The study was mainly evaluated through two representative cross-sectional surveys in random samples of NHD households: a baseline survey in 2/2006 and a follow-up survey in 2/2007. In addition, a qualitative study on community perceptions regarding the interventions was conducted in mid-2007 and data on costs were collected continuously to produce a detailed comparative cost analysis (these data will be published separately).

The primary outcomes of the study were overall *Serena* ITN ownership in households (total number of households with at least one observed *Serena* ITN, and total number of observed *Serena* ITNs). Secondary outcomes were overall bed net and ITN ownership of households, total number of bed nets, and bed net use. In order to demonstrate a 15% difference in ITN household ownership between intervention A and B and applying the design factor 2 to account for potential inter-cluster correlation, the required sample size was 500 households per study arm [39]. From each cluster, 20 households were selected from the village where the health centre is situated and another 20 households from one randomly chosen villages of the catchments area of the health centre. Households in respective villages were selected using a modification of EPI cluster sampling methodology [40]. As we did not aim to provide population-based ITN ownership estimates, household sampling was not done proportional-to-size of respective villages. Only in the town of Nouna, to take into account the much larger population, we purposely selected 70 instead of 20 households.

In February 2006 and 2007 respectively, a specific team from the *Centre de Recherche en Santé de Nouna* (CRSN) conducted the surveys in the selected study villages. The questionnaire, which was exactly the same across the two years, was pre-tested in two villages and comprised questions on the socio-demographic characteristics of the household, on pregnancy, on bed net and specifically ITN numbers, type and ownership, and on bed net use during last night and preceding rainy season. The household head answered all questions on the socio-demographic characteristics of the household. After his/her permission was obtained, the single users, or in case of children their main caregiver, were asked information on bed net ownership, type and use. The following types of bed nets were encountered: a.) various types of traditional non-impregnated bed nets, b.) ITNs from an earlier study (green colour), c) *Serena* ITNs (white colour, similar for intervention A and B). Pregnant women themselves answered questions on pregnancy.

### Statistical analysis

Field data forms were checked manually by supervisors for completeness before independent computer entry (Microsoft ACCESS, version 97) at CRSN. All data were checked for range and consistency. Any differences were resolved by checking against the original case record forms.

Descriptive tables are given to present bed net and ITN ownership by groups of interest. Prevalence in intervention and control group was always compared with exact Fischer-Test. All analyses were done with the statistical software package SAS (version 9.1).

### Ethical aspects

Approval was granted by the Ethical Committee of the Heidelberg University Medical School and the local Ethical Committee in Burkina Faso. Prior to the study implementation, the trial was explained in detail to and discussed with all district authorities and the District Health Team including all nurses of the NHD, as well as with the project team of PSI. During the two surveys, oral informed consent was sought from the respective heads of households and household members who were interviewed.

### Role of funding sources

The sponsor of the study had no role in study design, data collection, data analysis, data interpretation, or writing of the report. The corresponding author had full access to all data in the study and had final responsibility for the decision to submit for publication.

## Results

### Characteristics of study households and participants

A total of 1052 households (480 group A, 572 group B) were interviewed during the baseline survey and 1050 households (480 group A, 570 group B) during the follow-up survey. The demographic and socio-economic characteristics of participating households are shown in table 1.

**Table 1:** Demographic and socio-economic characteristics of households and household heads participating in the baseline and follow-up surveys.

	Baseline (2/2006)			Follow-up (2/2007)		
	A	B	*p* (A vs. B)	A	B	*p* (A vs. B)
**General characteristics**						
**No of HH**	480	572		480	570	
**Median no of rooms (range)**	3 (1–28)	3 (1–29)	n.s.	3 (1–19)	3 (1–23)	n.s.
**No HH with electricity (%)**	2 (0.4)	23 (4.0)	<0.001	4 (0.8)	31 (5.4)	<0.001
**No HH with water pipes (%)**	0 (0)	3 (0.5)	n.s.	1 (0.2)	0 (0)	n.s.
**No of inhabitants (mean/HH)**	4426 (9.2)	5539 (9.7)	n.s.	4103 (8.5)	5027 (8.8)	n.s.
**No of children<5 ys (%)**	830 (18)	1048 (19)	n.s.	771 (19)	979 (19)	n.s.
**No of pregnant women (%)**	72 (1.6)	100 (1.8)	n.s.	107 (2.6)	105 (2.1)	n.s.
**Head of HH characteristics**						
**Male (%)**	456 (95)	546 (95)	n.s.	447 (93)	541 (95)	n.s.
**Ethnicity (%)**						
**Bwaba**	246 (51)	193 (34)	<0.001	250 (52)	200 (35)	<0.001
**Marka**	132 (28)	170 (30)	n.s.	139 (29)	180 (32)	n.s.
**Mossi**	51 (11)	40 (7)	0.047	46 (10)	32 (6)	0.018
**Peulh**	36 (8)	66 (12)	0.028	37 (8)	68 (12)	0.023
**Samo**	7 (1)	42 (7)	<0.001	7 (1)	44 (8)	<0.001
**Other**	7 (1)	61 (11)	<0.001	1 (0.2)	46 (8)	<0.001
**Religion (%)**						
**Muslim**	195 (41)	332 (58)	<0.001	194 (40)	339 (59)	<0.001
**Catholic**	149 (31)	149 (26)	n.s.	151 (31)	144 (25)	0.028
**Protestant**	59 (12)	28 (5)	<0.001	57 (12)	32 (6)	<0.001
**Animist**	73 (15)	62 (11)	0.041	77 (16)	55 (10)	0.002
**Other**	1 (0.2)	1 (0.2)	n.s.	1 (0.2)	0	n.s.
**Literacy (%)**						
**Formal school**	61 (13)	84 (15)	n.s.	70 (15)	94 (16)	n.s.
**Koran school**	47 (10)	84 (15)	0.019	32 (7)	70 (12)	0.002
**Alphabetic course**	47 (10)	40 (7)	n.s.	86 (18)	47 (8)	<0.001
**Marital status (%)**						
**Single**	8 (2)	3 (1)	n.s.	1 (0.2)	5 (1)	n.s.
**Monogamous marriage**	313 (65)	388 (68)	n.s.	302 (63)	382 (67)	n.s.
**Polygamous marriage**	125 (26)	142 (25)	n.s.	136 (28)	143 (25)	n.s.
**Divorced**	10 (2)	3 (1)	0.026	4 (1)	7 (1)	n.s.
**Widowed**	24 (5)	36 (6)	n.s.	37 (8)	33 (6)	n.s.

HH = household; No = number; ITN = insecticide-treated bed net; A = intervention A (social marketing program of ITNs+free distribution of ITNs to pregnant women in health centres); B = intervention B (social marketing program of ITNs); information on ethnicity and religion was missing in a few cases; n.s. = non significant (p<0.05).

The mean number of household members was 9.1 living on average in three rooms, with about one fifth being children under the age of five years. There was no statistically significant difference between group A and B in the number of pregnant women at baseline (1.6% and 1.8%, p = 0.50), but a tendency towards more pregnant women in group A compared to group B at follow-up (2.6% vs 2.1%, p = 0.11). Overall, very few households had water pipes or electricity; most of these were situated in area B which includes Nouna town.

The typical head of household was male, illiterate, monogamous married, of Bwaba or Marka ethnicity, and of Muslim or Christian religion. There were some differences in the distribution of ethnicity, religion and literacy between group A and group B, but household heads were rather similar with regard to marital status.

### Efficacy of the intervention

The effects of the ITN intervention on outcome measures are given in table 2–5.

**Table 2:** Effects of the intervention on bed net, ITN and *Serena* ITN household ownership by study group.

	Baseline (2/2006)			Follow-up (2/2007)		
	A	B	*p* (A vs. B)	A	B	*p* (A vs. B)
**No of HH with at least one ** ***Serena*** ** ITN (%)**	64/480 (13)	102/572 (18)	n.s.	167/479 (35)	131/570 (23)	<0.001
**No of HH with at least one ** ***Serena*** ITN (%)*	64/480 (13)	75/482 (16)	n.s.	167/479 (35)	97/482 (20)	<0.001
**No of HH with at least one ITN (%)**	100/480 (21)	143/572 (25)	n.s.	207/479 (43)	177/570 (31)	<0.001
**No of HH with at least one bed net (%)**	246/480 (51)	326/572 (57)	n.s.	293/479 (61)	333/570 (58)	n.s.

HH = household; No = number; ITN = insecticide-treated bed net; A = intervention A (social marketing program of ITNs+free distribution of ITNs to pregnant women in health centres); B = intervention B (social marketing program of ITNs);^*^Nouna town cluster taken out of the analysis; n.s. = non significant (p<0.05).

**Table 3 pone-0003182-t003:** Effects of the intervention on the number and types of bed nets by study group.

	Baseline (2/2006)			Follow-up (2/2007)		
	A	B	*p* (A vs. B)	A	B	*p* (A vs. B)
**Total no of bed nets**	528	722		636	775	
***Serena*** ** ITNs**	99	160	n.s.	249	224	<0.001
**ITNs (earlier study)**	56	64	n.s.	59	79	n.s.
**Other bed nets**	354	466	n.s.	326	465	0.001
**Bed net type not clear**	14	19	n.s.	1	4	n.s.
**Total no of ** ***Serena*** ** ITNs/total no of bed nets (%)**	99/528 (19)	160/722 (22)	n.s.	249/636 (39)	224/775 (29)	<0.001
**Total no of ITNs/total no of bed nets (%)**	155/528 (29)	224/722 (31)	n.s.	308/636 (48)	303/775 (39)	<0.001

HH = household; No = number; ITN = insecticide-treated bed net; A = intervention A (social marketing program of ITNs+free distribution of ITNs to pregnant women in health centres); B = intervention B (social marketing program of ITNs); information on type of bed net was missing in a few cases; n.s. = non significant (p<0.05).

**Table 4 pone-0003182-t004:** Origin of bed nets by study group at baseline and follow-up.

	Baseline (2/2006)			Follow-up (2/2007)		
	A	B	*p* (A vs. B)	A	B	*p* (A vs. B)
**Bought at shops/markets**	393	588	0.003	415	618	<0.001
**Present of family/friend**	70	47	<0.001	43	62	n.s.
**Free distribution (earlier study)**	64	77	n.s.	71	90	n.s.
**Free distribution through ANC**	1	0	n.s.	103	3	<0.001
**Selfmade**	0	9	0.013	3	0	n.s.
**Total no of bed nets**	528	721	<0.001	635	773	<0.001

HH = household; ANC = antenatal care service in health centre; No = number; ITN = insecticide-treated bed net; A = intervention A (social marketing program of ITNs+free distribution of ITNs to pregnant women in health centres); B = intervention B (social marketing program of ITNs); information on bed net origin was missing in a few cases; n.s. = non significant (p<0.05).

**Table 5:** Bed net use (bed nets are partly used by more than one person) by study group at baseline and follow-up.

	Baseline (2/2006)			Follow-up (2/2007)		
	A	B	*p* (A vs. B)	A	B	*p* (A vs. B)
**Last night use of bed net (%)**	157/528 (30)	236/722 (33)	n.s.	214/636 (34)	306/775 (39)	0.027
**By head of HH**	32	70		52	83	
**By women**	74	116		118	144	
**By pregnant women**	5	5		10	14	
**By young children**	75	108		123	145	
**By other persons**	71	89		48	94	
**Last rainy season bed net use**						
**By head of HH**	109	184		149	194	
**By women**	229	311		261	340	
**By pregnant women**	44	44		44	44	
**By young children**	253	292		254	336	
**By other persons**	195	283		144	231	

HH = household; No = number; ITN = insecticide-treated bed net; A = intervention A (social marketing program of ITNs+free distribution of ITNs to pregnant women in health centres); B = intervention B (social marketing program of ITNs); n.s. = non significant (p<0.05).

Considering only *Serena* ITNs, household ownership increased significantly from 16% to 28% over the study period (166/1052 vs. 298/1049, p<0.001). *Serena* ITN household ownership increased significantly between baseline and follow-up in the area A (64/480 vs. 167/479, p<0.001) but not in area B (table 2). If the Nouna cluster was taken out of this analysis, the number of households reporting the possession of at least one *Serena* ITN in intervention area B increased from 75/482 (16%) to 97/482 (20%). This did not change the statistically significant difference between the number of households reporting the possession of at least one *Serena* ITN in intervention area A compared to B at follow-up (table 2).

Total household bed net ownership increased significantly from 54% to 60% (572/1052 vs. 626/1049, p<0.001) over the study period (table 2). At baseline, there were more but not significantly more households reporting the possession of at least one bed net in intervention area B compared to A (326/572 vs 246/480); if the Nouna cluster was taken out of this analysis, these figures changed to 261/482 vs. 246/480. At follow-up, the number of households reporting the possession of at least one bed net increased in both intervention areas, but the increase was only significant in the intervention area A (246/480 vs. 293/479, p = 0.002). If the Nouna cluster was taken out of this analysis, the number of households reporting the possession of at least one bed net in intervention area B increased from 261/482 (54%) to 279/482 (58%), which is not statistically significant.

The overall number of bed nets, ITNs and *Serena* ITNs increased in both intervention areas over time, but this was more marked in intervention area A compared to B. In addition, a number of ITNs were observed in both intervention areas which originate from an ITN trial with individual randomisation carried out since 2002 in 41 villages close to Nouna town [12]. Six clusters of this study were located in the CRSN ITN study area (three in area A and B respectively). These ITNs were recognised of being different from the *Serena* ITNs both by the interviewers and the respondents through their different colour (green) and because they have been distributed a few years earlier through specific field staff of the CRSN. Considering also these ITNs, the proportion of bed nets being ITNs reached nearly 50% during the follow-up (table 3).

The great majority of existing bed nets in households was bought in shops or at markets, with a significant increase over time (981/1249 vs. 1033/1408, p = 0.002). Some bed nets were reportedly donated by friends or relatives and some resulted from an earlier trial with a different type of ITN in a sub-area of NHD. Free distribution of *Serena* ITNs through the health centres was nearly exclusively reported from the area A during the follow-up survey (table 4).

The number of bed nets reported to have been used to protect household members in the night before the interview was 393/1250 (31%) at baseline and increased to 520/1411 (37%) during follow-up, without major differences between the two intervention groups. Bed nets were reported to have been used in the last night by different household members including the head of household, women, and children, and often by more than one person. The number of bed nets reported to have been used to protect household members was much higher during the rainy season compared to the dry season (table 5). The proportion of *Serena* ITNs having been used the night before by pregnant women and/or children below 5 years was 44/99 (44%) in group A and 59/160 (37%) in group B at baseline and increased to 125/249 (50%) in group A and 99/224 (44%) in group B at follow-up.

## Discussion

This study reports data from a large community-based cluster-randomised controlled trial on different ITN distribution systems conducted in a typical province of rural West Africa. The study area in Burkina Faso is highly endemic for malaria and mainly populated by farmers living from subsistence agriculture. The degree of underdevelopment is illustrated by a high illiteracy rate as well as by a very low rate of electricity and piped water in the participating households.

The study evaluated the effects of two interventions: social marketing of ITNs compared to social marketing of ITNs combined with free distribution of ITNs to all pregnant women attending antenatal care services in governmental health centres. Social marketing of the *Serena* ITNs through PSI took already place in Burkina Faso since a couple of years but had concentrated so far mainly on the big cities. The fact, that the ITN social marketing arm of this study was carried out through PSI, who applied the same approach that is used in the country already for some time, and that the ITN free distribution arm was carried out as a national pilot programme through the District Health Team of NHD, demonstrates, that the study was implemented under real programme conditions.

The main finding from the study is an increase of ITN ownership over time, which is mainly attributed to a significantly higher increase of *Serena* ITN ownership in the area where social marketing and free distribution through health services were combined compared to the areas with social marketing only. That this effect is largely due to the free distribution of ITNs is supported by the steep increase of bed nets obtained from the governmental antenatal care services over the study period. This was achieved despite the fact that *Serena* ITN ownership was higher at baseline in area B, which is explained by preceding *Serena* ITN sales in Nouna town, which belongs to intervention B.

That social marketing also played a role in the observed increase in bed net and ITN ownership is shown by the significant increase in bed net purchase over the study period. This phenomenon was observed in both intervention areas, which provides some evidence that free ITN distribution does not necessarily interfere with sales. However, as all *Serena* ITNs provided for social marketing were made availably in NHD already at the start of the programme, it is likely that a high proportion of these ITNs provided to the Nouna wholesaler were finally sold to other places of Burkina Faso or even to neighbouring Mali (personal observations). A significant leakage of highly subsidised ITNs, a product on high demand in many SSA communities, is not surprising and can be attributed to unsaturated markets surrounding the intervention province, as also shown in other SSA countries [41].

The main result from this study thus generally supports the idea that giving out ITNs through governmental health services free of charge could be a key component of sustainable ITN distribution systems in rural SSA [34]. Similar evidence was already reported from evaluations of the first national ITN programme in SSA [42], [43].

Reported last night use of bed nets was rather low during both surveys and also in comparison to reported bed net use in the previous rainy season, which is likely explained by the fact that both surveys took place in the middle of the dry season. This supports recent findings of low bed net use outside the rainy season from an ITN compliance study in young children living in the same area [38] as well as similar observations from other areas of SSA [44], [45].

Regarding bed net behaviour, the observed increase in use over the study period in the target groups of pregnant women and young children is promising, although there may well be ongoing problems with adults in households not respecting the goal of preferably protecting the primary malaria risks groups, as it has also been reported from other SSA countries [46]. Preferential protection of young children with ITNs in a sub-area of NHD was already achieved in a large efficacy study, pointing to the effects of clear instructions to the population [12], [38]. Thus, education campaigns accompanying ITN programmes should further reinforce the message that pregnant women and young children are at the highest risk for severe malaria and deaths.

An interesting observation during this study was the tendency of the number of pregnant women to increase in the area of intervention A compared to and probably at the expense of area B over time. However, as there was no corresponding increase in antenatal care attendance rate of pregnant women in area A compared to area B (data not shown), this is most likely a chance finding. The provision of free ITNs through antenatal care services to all pregnant women would significantly contribute to reductions in the rates of maternal morbidity, placental malaria and low birth weight children, and consequently reduce maternal and infant mortality [47]–[49]. Given the high coverage in most of SSA, antenatal care services provide a unique opportunity to reach these target groups [50]. Moreover, a mutual benefit may occur as the provision of ITNs through mother and child services is expected to strengthen these services and to give their counselling on malaria prevention more credibility. Finally, all newborn infants would automatically be protected against malaria while sleeping with their mother under the ITN.

Recently published studies have confirmed the large public health benefit of ITN programmes in malaria endemic countries. In Kenya, ITN coverage has increased rapidly from 7% in 2004 to 67% in 2006. This was achieved through a combined approach of social marketing and mass free distribution and translated into reductions in childhood mortality comparable with those seen in efficacy trials [51]. The Kenya experience clearly documented the importance of free ITN distribution for a rapid achievement of high and equitable coverage [52]. As a consequence, a new position statement of the World Health Organisation calls for large scale free distribution of LLIN in endemic countries through health services accompanied by other distribution and sensitization campaigns [53]. This statement is also supported by the documented success of ITN distribution integrated with outreach campaigns (measles and polio immunisation, Vit A and anti-heminthic medication distribution) in SSA countries such as Zambia, Togo and Niger [44], [45], [54].

This study has some limitations. Firstly, although the study groups were quite comparable with regard to baseline characteristics, there were also some differences which could have confounding effects, for example in the distribution of ethnicity and religion. Moreover, the selection of one of the two villages in each cluster being the village the health clinic was in, as well as use of the EPI cluster method for household selection, could have lead to falsely elevate estimates of coverage. Secondly, the study was not based on a national programme and this could have influenced the supposed leakage of *Serena* ITNs. It is likely that a certain amount of the ITNs was sold to places outside the study province, as there was and still is a high demand for subsidised ITNs in Burkina Faso. Thirdly, the study had only two arms and not a third arm with only free ITN distribution through antenatal care services. The latter option would have meant to exclude a part of the study area from an established national intervention, posing mayor ethical as well as operational issues as well creating a very artificial scenario. Thus, the independent contribution of the different intervention approaches has not been fully assessed. Finally, the Nouna town cluster which has been assigned to the social marketing only intervention could have introduced a bias; thus it was reassuring that an analysis without the Nouna town cluster has arrived at rather similar results. However, despite these limitations we believe that this study provides important findings which help to judge the comparative benefits of two major ITN distribution channels in the real world of rural SSA.

In conclusion, the addition of free distribution of ITNs through ANC services to a social marketing program dramatically improved ITN ownership in the areas in which it was implemented. While ITN ownership increased somewhat in the social marketing only arm, with that rate of rise, it would take many years to reach the Roll Back Malaria targets. The addition of free ITN distribution and the added rate of rise may dramatically decrease the number of years required to reach global coverage goals and ultimately decrease malaria morbidity and mortality sooner.

## Supporting Information

Checklist S1 CONSORT checklist.
(0.06 MB DOC)

Protocol S1 Trial Protocol.
(0.22 MB DOC)
