# Supplementary material for: Distribution Systems of Insecticide-Treated Bed Nets for Malaria Control in Rural Burkina Faso: Cluster-Randomized Controlled Trial
Source: PLoS One. 2008 Sep 11;3(9):e3182. doi: 10.1371/journal.pone.0003182 (PMC2527521; doi:10.1371/journal.pone.0003182)
Supplement: Protocol S1 — Trial Protocol. (0.22 MB DOC) [file pone.0003182.s002.doc]

## General information about the project section D4

### 3.1.1 Title:

Community-effectiveness of the distribution of insecticide-treated bed nets through social marketing antenatal care services in malaria control in rural Burkina Faso

Gemeinde-Effektivität von über Schwangerenberatungsstellen verteilten Insektizid-imprägnierten Mosquitonetzen bei der Bekämpfung der Malaria im ländlichen Burkina Faso

### 3.1.2 Research areas and direction:

### Public Health

###

### 3.1.3 Project Section Leader:

Müller, Olaf; 05.12.1954

Ruprecht-Karls-Universität, Klinikum, Hygiene-Institut,

Abteilung Tropenhygiene und Öffentliches Gesundheitswesen,

Im Neuenheimer Feld 324, 69120 Heidelberg

Tel.: 0049-6221-56-5035

Fax: 0049-6221-56-5948

E-mail: [olaf.mueller@urz.uni-heidelberg.de](mailto:olaf.mueller@urz.uni-heidelberg.de)

Is the post held by the project leader for a fixed period?

No  Yes, until 6/2010

Further employment is planned

Jahn, Albrecht; 20.09.1954

Ruprecht-Karls-Universität, Klinikum, Hygiene-Institut,

Abteilung Tropenhygiene und Öffentliches Gesundheitswesen,

Im Neuenheimer Feld 324, 69120 Heidelberg

Tel.: 0049-6221-56-5607

Fax: 0049-6221-56-5037

E-mail: [albrecht.jahn@urz.uni-heidelberg.de](mailto:albrecht.jahn@urz.uni-heidelberg.de)

Is the post held by the project leader for a fixed period?

No  Yes, until _________________

Further employment is planned until ____________

### Within the project section it is intended to:

- carry out research on human subjects  Yes  No
  A copy of the required approval of the responsible ethics committee is included
- conduct clinical studies in
  somatic gene therapy  Yes  No
- conduct experiments involving animals  Yes  No
- conduct experiments involving
  recombinant DNA  Yes  No
- carry out research with human embryonic
  stem cells  Yes  No
- The legal authorisation has been obtained  Yes  No

## 3.2 Summary

The hypothesis that insecticide-treated bed net (ITN) effects may not be long-lasting in young children living in areas of intense malaria transmission due to interactions with the immunologi-cal development has now been refuted in a number of studies including the D4 study. The highly controversial question remains how African programmes can best reach a sustainable high coverage with ITNs in young children and pregnant women. Against this background it is planned to implement a cluster randomised controlled trial in Nouna Health District in Burkina Faso. Twenty-two peripheral health centres and their catchment areas will be randomised to (1) ITN provision to the general population through social marketing and (2) ITN provision to the general population through social marketing plus free provision to all pregnant women through antenatal services. The primary outcomes are ITN coverage in households and ITN use during pregnancy and infancy.

**Zusammenfassung**

Die Hypothese, dass Insektizid-behandelte Mosquitonetze (ITN) bei Kleinkindern in Gebieten hoher Malariaendemizität langfristig nicht effektiv sein könnten, wurde in den letzten Jahren von verschiedenen Studien einschliesslich der D4 Studie widerlegt. Als hochkontrovers diskutiertes Thema bleibt allerdings die Frage, wie am besten auf Programmebene ein nachhaltig hoher Schutz mit ITN für Kleinkinder und schwangere Frauen in afrikanischen Malariagebieten erreicht werden kann. Vor diesem Hintergrund planen wir eine Gruppen-randomisierte kontrollierte Studie im Nouna Gesundheitsdistrikt in Burkina Faso. Es ist eine Randomisierung des Einzugsgebietes von 16 ländlichen Gesundheitsstationen vorgesehen zu (1) Ausgabe von ITN an alle schwangeren Frauen über Schwangerenvorsorge-Dienste und (2) ITN Ausgabe an die Allgemeinbevölkerung über ein Social Marketing System. Hauptendpunkte der Studie sind die Versorgung der Haushalte mit ITN und die Benutzung von ITN während der Schwangerschaft und zum Schutz der Säuglinge und Kleinkinder.

## 3.3 Report on the development of the project section thus far

##

## 3.3.1 Report

**Scientific background for studies during phase 1 and 2 of SFB 544**

## Insecticide-treated nets (ITN) and particularly mosquito nets as a new tool in malaria control have received considerable interest over the last two decades. During the 1980s, a number of small-scale studies with synthetic pyrethroid insecticides have demonstrated significant reductions in malaria morbidity associated with ITN use in different African, Asian and Latin-American populations (Lindsay & Gibson 1988; Rozendaal 1989; Bermejo & Veeken 1992). Subsequently, a number of large randomised-controlled ITN efficacy and effectiveness trials have been conducted during the 1990s in sub-Saharan Africa (SSA). These trials have consistently demonstrated the efficacy and partly also the effectiveness of ITNs in reducing malaria morbidity and mortality over a broad range of malaria transmission intensities (Lengeler 1998; Phillips-Howard *et al.* 2003). However, as all these trials were only designed for short intervention periods, the long-term consequences of particularly young children’s protection from malaria through ITNs were not known. The value of using such an intervention in areas with high malaria transmission intensity had been discussed very controversially in the recent past. The major scientific questions regarding the use of ITNs for the protection of young children concerned the interaction between passively acquired immunity and ITN use during the first months of infancy as well as a possible shift of malaria morbidity and mortality to older age groups (Snow et al. 1995, Trape et al. 1996, Snow et al. 1997, Alles 1998). The debate remained controversial over the following years with various publications on this subject (Mondiano et al. 1998, Beier et al. 1999, Thomas & Lindsay 2000; Smith et al. 2001, Mathanga & Molyneux 2001, Armstrong Schellenberg et al. 2001, Coleman et al. 2001, Abdulla et al. 2001, Binka et al. 2002, Maxwell et al. 2002).

#### *Study-objectives, design and methods*

The main objective of the D4 trial was to determine differences in the rates of malaria morbidity and mortality as well as all-cause mortality in children protected with ITNs from birth up to their fifth birthday (group A) compared to children protected with ITNs from month seven up to their fifth birthday (group B) in a holoendemic area of West Africa.

The study was designed as an individually randomized controlled ITN community trial (n=3.400). Newborn children were recruited for the study from June 2000 until December 2002 from all 39 villages of the study area of the Centre de Recherche en Santé de Nouna (CRSN) in Nouna Health District in north-western Burkina Faso. Mothers and their children were provided at the time of delivery (group A) or after six months (group B) with deltamethrin-treated mosquito nets (PermaNet) through field staff regularly visiting the study villages. The primary study endpoints were all-cause mortality in cohort children measured through the existing demographic surveillance system (DSS) of the Nouna Health Research Centre in the study region, and malaria morbidity measured through bi-annual cross-sectional surveys and longitudinal follow-up of malaria incidence in sentinel villages.

### *Results*

- In preparation for this study, a survey of traditionally existing mosquito net coverage in the study area has been carried out in 1998. Around 35% of households were reporting ownership of at least one mosquito net, with high heterogeneity with regard to village and ethnic background (Traoré et al. 2002, Traoré 2003).
- Qualitative and quantitative anthropological research carried out during 1999 has guided the selection of mosquito nets for the ITN trial. The majority of mothers preferred rectangular big size synthetic bed nets of dark colour. Thus the project has bought rectangular family-size dark-green mosquito nets, pre-impregnated with Deltamethrin (first generation PermaNet) (Müller et al. 2002).
- An anthropological study on community factors associated with malaria prevention by mosquito nets revealed traditional concepts and practices in the Nouna area. Mosquito nets were confirmed to be prevalent in the villages, but mainly used by adults against mosquito nuisance (Okrah et al. 2002).
- In the research zone of the Nouna Health Research Centre, malaria in young children is mainly caused by *P. falciparum*. The transmission intensity is high and seasonal, reaching several hundred infectious bites per person per year, but with major differences between villages (Traoré 2003, Müller 2001, Müller et al. 2001)
- Corresponding childhood mortality and malaria-specific mortality is high in the area, with again major differences between villages (Sankoh et al. 2001, Müller et al. 2003a). Anaemia is the major manifestation of severe falciparum malaria in the study area, with malnutrition being an important co-factor (Müller et al. 2003b).
- Most fever/malaria treatment in the Nouna research zone is home treatment with chloroquine, antipyretics and traditional remedies (Müller et al. 2004a, Müller et al. 2003c).
- Resistance against chloroquine, the first-line treatment for uncomplicated malaria, is already prevalent in the CRSN study area (Müller et al. 2003d). In contrast, pyrimethamine-sulfadoxine is still sufficiently effective (Müller et al. 2004b)
- Results from a joint study of SFB 544 groups A1 and D4 are pending. Serum samples were collected over a period of 12 months from 120 infants of the D4 study in 2001/2002. These sera are currently processed in Heidelberg with a specific gp190 ELISA developed by Prof. Bujard.
- A study on antenatal care (ANC) coverage and malaria prevention behaviour in pregnant women in the rural CRSN study area demonstrated a sufficient ANC coverage and to good compliance with malaria and anaemia prevention measures (Mene Miaffo 2004).
- An efficacy evaluation of a prototype long-term impregnated ITN (PermaNet) has shown that this first-generation product was not much superior to conventional ITN, but that it can successfully be retreated in the field (Müller et al. 2002, Müller et al. 2004c).
- A study on behavioural determinants of compliance with the ITN intervention in 2003 showed that compliance was very good during the rainy season (99%) and moderate during the dry season (70%) in the CRSN study area (Frey, PhD thesis, unpublished).
- Preliminary data analysis of the major outcomes from the ITN trial point to:

a) A high demand and acceptance of ITN in rural Burkina Faso

b) No adverse events associated with exposure to ITN in young infants

c) No differences between group A and B in all-cause mortality (121 versus 117 deaths after 2.5 years of follow-up)

d) Significant and sustained malaria morbidity reduction in group A compared to group B after 2.5 years of follow-up (relative risk for falciparum malaria episodes 3.1 in young infants and 1.1 in the whole cohort) (Müller et al. 2003e, Müller et al., submitted)

*Conclusions*

- - ITN are safe in infants and young children living in areas of high malaria transmission intensity
  - ITN are more effective against malaria when protection starts already in early infancy

**Scientific background for studies planned during phase 3 of SFB 544**

ITNs were already employed on a large-scale since the 1980s in many malaria endemic areas of Asia, where they have contributed to major successes in malaria control (Hung *et al.* 2002). Today, the need for a large scale introduction of ITNs in SSA is well accepted in the scientific community as well as among international agencies (Curtis *et al.* 2003; Lines *et al.* 2003; Whitty *et al.* 2002; Hawley *et al.* 2003; WHO 2002). A high coverage with ITN would also lead to a mass effect on the mosquito populations similar to what can be achieved through systematic insecticide spraying programmes (Binka et al. 1998, Ilboudo-Sanogo et al. 2001, Maxwell et al. 2002). However, due to major differences in infrastructure, public service organisation, funds and epidemiology, progress in the implementation of ITN intervention in SSA was slow (Victora et al. 2004). Today, only some 2% of children in SSA are protected with ITN (WHO 2003).

So what is the best strategy for scaling-up ITN coverage in SSA? Two approaches – often seen as incompatible – are competing with each other in the international debate. The first group argues to consider ITN as a public good like vaccines and to consequently provide them through the public sector free of charge, while the second group argues for strengthening commercial markets but acknowledges the importance of subsidies for the groups most at risk (e.g. pregnant women and young children) (Curtis *et al.* 2003; Lines *et al.* 2003). The group in favour of free ITN distribution and re-treatments supports their argumentation by the evidence from a few east African pilot projects regarding the feasibility of such an approach, the existence of a significant community effect in most areas with high ITN coverage, the reality of a high proportion of the rural populations in SSA being unable to pay for such an intervention, and the hope that rich countries would be willing to pay for malaria control in SSA (Curtis *et al.* 2003). On the other side, the group in favour of strengthening commercial markets supports their argumentation by the success of a large ITN social marketing programme in rural Tansania (Armstrong-Schellenberg *et al.* 1999; Hansen *et al.* 2003; Mushi et al. 2003), the important role of market involvement in the success of ITN programmes in Asia, the assumption that free ITN provision would destroy local commercial markets, and the uncertainty of a continuous availability of external funds for ITN programmes. There is however considerable agreement in both groups regarding the need for major donor assistance for whatever approach (e.g. through the Global Fund for AIDS, malaria and Tb) (Curtis *et al.* 2003; Lines *et al.* 2003).

We have recently proposed an alternative approach for expanding ITN coverage in SSA (Müller and Jahn 2003). As pregnant women and young children are clearly the two groups most at risk for malaria morbidity and mortality in most of SSA, we believe that the focus of ITN programmes should be on these two population groups. The provision of free ITNs through antenatal care (ANC) services to all pregnant women would significantly contribute to reductions in the rates of maternal morbidity, placental malaria and low birth weight children, and consequently reduce maternal and infant mortality (Dolan *et al.* 1993; D`Alessandro *et al.* 1996; Ter Kuile *et al.* 2003). Given the high coverage in most of SSA, ANC provides a unique opportunity to reach these target groups (Hamel *et al.* 2001). Moreover, the provision of ITN would likely attract more women to ANC services and may thus provide additional health benefits to this important target group (Fraser-Hurt & Lyimo 1998). Moreover, a mutual benefit may occur as the provision of ITNs through Mother and Child services is expected to strengthen these services and to give their counselling on malaria prevention more credibility. After birth, all infants would automatically be protected against malaria while sleeping with their mother under the ITN.

What about ITN re-impregnation with insecticide? We know that re-impregnation is needed in certain intervals due to loss of insecticide over time, which is mainly influenced by washing frequency (Lines 1996a). Thus, re-impregnation services will also need to be provided to assure a continuous protection of children throughout their early life and until they have developed their own immunity. Such services should also be free of charge since otherwise coverage will be very low as demonstrated in many ITN projects in SSA (Lines *et al.* 1996b; Cham *et al.* 1997; Armstrong-Schellenberg *et al.* 1999). Technical progress has now enabled the development of reliable long-lasting insecticide-treatment, thus re-treatment services may no longer be needed (Gonzales et al. 2002, Kröger et al. 2003, Kilian – personal information, Lengeler – personal information). However, it remains to be demonstrated that these long-lasting ITNs really last for the entire lifetime of a mosquito net under the circumstances of different communities in SSA.

What is the evidence for providing pregnant women and subsequently their young children with free ITN and free re-treatment being feasible and effective. Initial evidence for this stems from the Gambian National Impregnated Bednet Programme (D`Alessandro *et al.* 1995). During the first year of this programme villagers were offered insecticide free of charge through their respective village health workers, while during the second year the insecticide was started to be sold at a prize of some 0.5 USD. The introduction of fees for service was accompanied by a sharp drop in impregnation coverage from 85% during the first year to only 14% in the second year (D`Alessandro *et al.* 1995; Cham *et al.* 1997). Cost-recovery was introduced into the whole country during the third year of the programme, and communities were offered insecticide again through village health workers but alternatively also through shops. In addition, women attending selected mother and child health (MCH) services were offered insecticide free of charge. An evaluation of the activities of the national programme during its third year provided evidence for offering insecticide through private channels leading to increased impregnation coverage, as well as for the provision of insecticide through MCH services being effective in targeting young children (Müller *et al.* 1997). These findings are furthermore supported by our experience from the ongoing D4 ITN trial in rural Burkina Faso, where all newborns and their mothers have been provided with free ITN and free annual re-impregnation (Müller *et al.* 2003e). This trial is now into its fourth year and coverage and re-impregnation rates have remained above 90%. As reported from other pilot projects with free provision of ITN, we also have no indication for ITN being sold or diverted (Curtis *et al.* 2003).

**References**

1. Abdulla S, Armstrong Schellenberg J, Nathan R, *et al.* Impact on malaria morbidity of a programme supplying insecticide treated nets in children aged under 2 years in Tanzania: community cross sectional study. *British Medical Journal*, 322: 270-273 (2001)

2. Alles. Malaria mortality rates in South Asia and in Africa: implications for malaria control. *Parasitology Today*, 14: 369-375 (1998)

3. Armstrong-Schellenberg J, Abdulla S, Minja H, *et al.* KINET: a social marketing programme of treated nets and net treatment for malaria control in Tanzania, with evaluation of child health and long-term survival. *Transactions of the Royal Society of Tropical Medicine and Hygiene* 93: 225-31 (1999)

4. Armstrong Schellenberg JRM, Abdulla S, Nathan R, *et al.* Effects of large-scale social marketing of insecticide-treated nets on child survival in rural Tanzania. *The Lancet,* 357: 1241-1247 (2001)

5. Beier JC, Killeen GF, Giture JI. Entomological inoculation rates and falciparum malaria prevalence in Africa. *American Journal of Tropical Medicine and Hygiene*, 61: 109-113 (1999)

6. Bermejo A & Veeken H. Insecticide-impregnated bed nets for malaria control: a review of the field trials. *Bulletin of the World Health Organisation* 70: 293-96 (1992)

7. Binka FN, Indome F, Smith T. Impact of spatial distribution of permethrin-impregnated bed nets on child mortality in rural Northern Ghana. *American Journal of Tropical Medicine and Hygiene*, 59: 80-85 (1998)

8. Binka FN, Hodgson A, Adjuik M, Smith T. Mortality in a seven-and-a-half-year follow-up of a trial of insecticide-treated mosquito nets in Ghana. *Transactions of the Royal Society of Tropical Medicine and Hygiene* 96: 597-99 (2002)

9. Cham MK, Olaleye B, D'Alessandro U, *et al.* The impact of charging for insecticide on the Gambian National Impregnated Bednet Programme. *Health Policy and Planning* 12: 240-47 (1997)

10. Coleman PG, Perry BD, Woolhouse MEJ. Endemic stability – a veterinary idea applied to human public health. *The Lancet,* 357: 1284-1286 (2001)

11. Curtis C, Maxwell C, Lemnge M, *et al.* Scaling-up coverage with insecticide-treated nets against malaria in Africa: who should pay? *The Lancet Infectious Diseases* 3, 304-7 (2003)

12. D`Alessandro U, Olaleye B, McGuire W, *et al.* Mortality and morbidity from malaria in Gambian children after an impregnated bed net programme. *The Lancet* 345: 479-83 (1995)

13. D`Alessandro U, Langerock P, Bennett S, *et al.* The impact of a national impregnated bed net programme on the outcome of pregnancy in primigravidae in The Gambia. *Transactions of the Royal Society of Tropical Medicine and Hygiene* 90, 487-92 (1996)

14. Dolan G, Ter Kuile FO, Jacoutot V, *et al.* Bed nets for the prevention of malaria and anaemia in pregnancy. *Transactions of the Royal Society of Tropical Medicine and Hygiene* 87: 620-26 (1993)

15. Fraser-Hurt N, Lyimo EO. Insecticide-treated nets and treatment service: a trial using public and private sector channels in rural United Republic of Tanzania. *Bulletin of the World Health Organisation* 76: 607-15 (1998)

16. Gonzales JO, Kroeger A, Avina AI, Pabon E. Wash resistance of insecticide-treated materials. *Transactions of the Royal Society of Tropical Medicine and Hygiene* 96:370-75 (2002)

17. Hamel MJ, Odhacha A, Roberts JM, Deming MS. Malaria control in Bungoma District, Kenya: a survey of home treatment of children with fever, bednet use and attendance at antenatal clinics. *Bulletin of the World Health Organisation* 79: 1014-23 (2001)

18. Hansen K, Kikumbih N, Armstrong-Schellenberg J, *et al.* Cost-effectiveness of social marketing of insecticide-treated mosquito nets for malaria control in the United Republic of Tanzania. *Bulletin of the World Health Organisation* 81: 269-76 (2003)

19. Hawley WA, Ter Kuile FO, Steketeee RS, *et al.* Implications of the western Kenya permethrin-treated bed net study for policy, program implementation, and future research. *The American Journal of Tropical Medicine and Hygiene* 68 (supplement): 168-73 (2003)

20. Hung LQ, de Vries PJ, Giao PT, *et al.* Control of malaria : a successful experience from Viet Nam. *Bulletin of the World Health Organisation* 80: 660-66 (2002)

21. Ilboudo-Sanogo E, Cuzin-Ouattara N, Diallo DA, et al. Insecticide-treated materials, mosquito adaptation and mass effect: entomological observations after five years of vector control in Burkina Faso. *Transactions of the Royal Society of Tropical Medicine and Hygiene* 95: 353-60 (2001)

22. Kroeger A, Skovmand O, Phan QC, Boewono DT. Combined field and laboratory evaluation of a long-term impregnated bednet, PermaNet. *Transactions of the Royal Society of Tropical Medicine and Hygiene* 98: 152-155 (2004)

23. Lengeler C. Insecticide treated bed nets and curtains for malaria control (Cochrane Review). In: The Cochrane Library, Issue 1, 2001. Oxford: Update Software (2001)

24. Lindsay SW & Gibson ME. Bednets revisited – old idea, new angle. *Parasitology Today* 4: 270-72 (1988)

25. Lines J. The technical issues. In Net Gain: A new method for preventing malaria deaths (editors Lengeler C, Cattani J, de Savigny D), World Health Organisation, ISBN 0-88936-792-2 (1996a)

26. Lines J. Mosquito nets and insecticides for net treatment: a discussion of existing and potential distribution systems in Africa. *Tropical Medicine & International Health* 1, 616-32 (1996b)

27. Lines J, Lengeler C, Cham K, *et al.* Scaling-up and sustaining ITN coverage in Africa. *The Lancet Infectious Diseases* 3: 466 (2003)

28. Mathanga D, Molyneux ME. Bednets and malaria in Africa. *The Lancet,* 357: 1219-1220 (2001)

29. Maxwell CA, Msuya E, Sudi M, *et al.* Effect of community-wide use of insecticide-treated nets for 3-4 years on malaria morbidity in Tanzania. *Tropical Medicine and International Health,* 7: 1003-8 (2002)

30. Mene Miaffo C, Somé F, Kouyaté B, Jahn A, Müller O. Malaria and anaemia prevention in pregnant women of rural Burkina Faso. *BMC Pregnancy and Childbirth*, 4: 18 (2004)

31. Mondiano D, Sirima BS, Sawadogo A, Sanou I, Paré J, Konaté A, Pagnoni F. Severe malaria in Burkina Faso: influence of age and transmission level on clinical presentation. *American Journal of Tropical Medicine and Hygiene*, 59: 539-542 (1998)

32. Müller O, Cham K, Jaffar S, Greenwood B. The Gambian National Impregnated Bednet Programme - evaluation of the 1994 cost recovery trial. *Social Science and Medicine* 44: 1903-1909 (1997)

33. Müller O, Becher H, Baltussen A, *et al.* Effect of zinc supplementation on malaria and other causes of morbidity in west African children: randomized double-blind placebo-controlled trial. *British Medical Journal,* 322: 1567-1572 (2001)

34. Müller O. Die Epidemiologie der Malaria in Nouna, Burkina Faso, West-Afrika. *Informatik, Biometrie und Epidemiologie in Medizin und Biologie*, 32: 235 (2001)

35. Müller O, Ido K, Traoré C. Evaluation of a prototype long-lasting insecticide-treated mosquito net under field conditions in rural Burkina Faso. *Transactions of the Royal Society of Tropical Medicine and Hygiene,* 96: 483-484 (2002)

36. Müller O, Garenne M, Kouyaté B, Becher H. The association between protein-energy malnutrition, malaria morbidity and all-cause mortality in West African children. *Tropical Medicine & International Health,* 8: 507-511 (2003a)

37. Müller O, Traoré C, Jahn A, Becher H. Severe anaemia in west African children: malaria or malnutrition?. *The Lancet,* 361: 86-87 (2003b)

38. Müller O, Traoré C, Kouyaté B, Becher H. Malaria morbidity, treatment seeking behaviour, and mortality in a cohort of young children in rural Burkina Faso. *Tropical Medicine & International Health,* 8: 290-296 (2003c)

39. Müller O, Traoré C, Kouyaté B. Clinical efficacy of chloroquine in young children with uncomplicated malaria – a community based study in rural Burkina Faso. *Tropical Medicine & International Health,* 8: 202-203 (2003d)

40. Müller O, Traoré C, Kouyaté B, Becher H. Effects of insecticide-treated mosquito nets (ITN) on malaria morbidity and all-cause mortality in infants of a malaria holoendemic area in rural Burkina Faso. *Acta Tropica,* 83 (supplement): 71-72 (2003e)

41. Müller O, Jahn A. Editorial: Expanding insecticide-treated mosquito net coverage in Africa: tradeoffs between public and commercial strategies. *Tropical Medicine & International Health,* 8: 853-56 (2003)

42. Müller O, Razum O, Traore C, Kouyate B. Community effectiveness of chloroquine and traditional remedies in the treatment of young children with falciparum malaria in rural Burkina Faso. *Malaria Journal* (2004a, in press)

43. Müller O, Traoré C, Kouyaté B. Efficacy of pyrimethamine-sulfadoxine in young children with falciparum malaria in rural Burkina Faso. *Malaria Journal,* 3: 10 (2004b)

44. Müller O, Frey C, Traoré C, Kouyaté B. Retreatment of long-lasting ITNs under field conditions in rural Burkina Faso. *Journal of Tropical Pediatrics,* 50: 6 (2004c)

45. Mushi AK, Armstrong Schellenberg JRM, Mponda H, Lengeler C. Targeted subsidy for malaria control with treated nets using a discount voucher system in Tansania. *Health Policy and Planning,* 18: 163-71 (2003)

46. Okrah J, Traoré C, Palé A, Sommerfeld J, Müller O. Community factors associated with malaria prevention by mosquito nets: an exploratory study in rural Burkina Faso. *Tropical Medicine and International Health,* 7: 240-248 (2002)

47. Phillips-Howard PA, Nahlen BL, Kolczak MS, *et al.* Efficacy of permethrin-treated bed nets in the prevention of mortality in young children in an area of high perennial malaria. *The American Journal of Tropical Medicine and Hygiene* 68 (supplement): 23-29 (2003)

48. Rozendaal JA. Impregnated mosquito nets and curtains for self-protection and vector control. *Tropical Diseases Bulletin* 86: 1-41 (1989)

49. Sankoh OA, Ye Y, Sauerborn R, Müller O, Becher H. Clustering of childhood mortality in rural Burkina Faso. *International Journal of Epidemiology,* 30: 485-492 (2001)

50. Smith TA, Leuenberger R, Lengeler C. Child mortality and malaria transmission intensity in Africa. *Parasitology Today*, 17: 145-149 (2001)

51. Snow RW. Will reducing P. falciparum transmission alter malaria mortality among African children? *Parasitology Today,* 11: 188-190 (1995)

52. Snow RW, Omumbo JA, Lowe B, *et al.* Relation between severe malaria morbidity in children and level of P. falciparum in Africa. *The Lancet*, 349: 1650-54 (1997)

53. Ter Kuile FO, Terlouw DJ, Phillips-Howard PA, *et al.* Reduction of malaria during pregnancy by permethrin-treated bed nets in an area of intense perennial malaria transmission in western Kenya. *The American Journal of Tropical Medicine and Hygiene* 68 (supplement), 50-60 (2003)

54. Thomas CJ, Lindsay SW. Local-scale variation in malaria infection amongst rural Gambian children estimated by satellite remote sensing. *Transactions of the Royal Society of Tropical Medicine and Hygiene*, 94: 159-63 (2000)

55. Traoré C, Becher H, Müller O. Epidemiology of malaria among young children in six villages of rural Burkina Faso. Oral presentation, III MIM Pan-African Malaria Conference, Arusha, November 2002

56. Traoré C. Epidemiology of malaria in a holoendemic area of rural Burkina Faso. PhD thesis, University of Heidelberg (2003)

57. Trapé JF. Combating malaria morbidity and mortality by reducing transmission. *Parasitology Today,* 12: 236-40 (1996)

58. Victora CG, Hanson K, Bryce J, Vaughan P. Achieving universal coverage with health interventions. *The Lancet*, 364: 1541-48 (2004)

59. Whitty CJM, Rowland M, Sanderson F, Mutabingwa TK. Science, medicine, and the future – Malaria. *BMJ* 325, 1221-24 (2002)

60. World Health Organisation. Scaling-up insecticide-treated netting programmes in Africa. Geneva: WHO/CDS/RBM/2002.43 (2002)

61. World Health Organisation. The Africa Malaria Report 2003. Geneva: WHO/CDS/MAL/2003.1093 (2003)

## 3.4 Planned continuation of the project section (objectives, methods, work programme)

**3.4.1 Aims**

**3.4.2 Methods**

Study design and definition of the intervention

This is a proposal for a community-based randomised controlled trial (RCT) with peripheral health centres (n=22) and their target villages as the units of randomisation. The catachment areas of the 22 health facilities will be randomised to receive either (1) ITN provision to the general population through social marketing (intervention A) or (2) ITN provision to the general population through social marketing plus free ITN provision to all pregnant women through antenatal services (intervention B). The National Malaria Control Programme (NMCP) is currently considering to shift its policy from one based exclusively on subsidised sales and social marketing to one which integrates free distribution among high-risk populations (i.e. pregnant women and young children).

Following a series of meetings in September 2005, it appears that the distribution of the ITNs will be secured by collaborative efforts between the NMCP, Population Services International (PSI), UNICEF, the Nouna Health District, the Nouna Health Research Centre (CRSN), and the Department of Tropical Hygiene and Public Health at the Heidelberg University (ATHÖG). Third generation PermaNet will be provided in both arms of the intervention.

Study outcomes

*Primary outcomes:* (1) ITN coverage in households 12 and 24 months after the start

of the interventions

(2) ITN use during pregnancy and infancy

*Secondary outcomes:* (1) Costs per malaria case and per DALY prevented

(2) Self-reported information on ANC visits

(3) Insecticide content on ITN and mortality of vector mosquitoes over time

(4) Acceptance of health staff and population

Sample size

The sample size calculation is based on the primary outcome ITN coverage. In order to demonstrate a 15% difference and applying the design factor 2 for clustering, the required sample size will be 400 households per study arm. In order to compensate for migration and possible loss in follow up, 500 households will be enrolled in each arm.

Study methods

By using standard two-stage EPI cluster sampling methodology, 500 randomly sampled households from the intervention A and 500 randomly sampled households from the intervention B area will be visited before the start of the intervention (February 2006). New random samples will be drawn again after 12 months and 24 months respectively. ITN coverage will be measured through self-reported information on a standardised questionnaire and through direct observation. ITN use during pregnancy and infancy will be measured through self-reported information on a standard questionnaire. These data will be validated through direct observations during the rainy season of 2007 in a sub-sample of 100 households in the study area. These households will be also part of the survey sample of January 2008.

Costing estimates and cost-effectiveness calculations will be undertaken in close collaboration with Prof. Dr. Steffen Flessa, University of Greifswald. Prof. Flessa has been a member of the Department of Tropical Hygiene and Public Health at the Heidelberg University until recently and is working on the costing of health interventions in Nouna. The current funding period of the SFB 544 has let project D7 in and Prof. Flessa will continue to work on costing of health intervention aspects in Burkina Faso after his move to Greifswald.

Random samples of 100 ITN will be taken at the time of the third household survey follow up in 2008, for determination of deltamethrin content using xylene extraction and capillary gas chromatography. In addition, ITN efficacy will be measured using standard WHO bioassays with the endpoint of mosquito mortality 24 hours after three minutes exposure to the ITN. The ITN washing frequency will be recorded.

Community perceptions regarding the different ITN distribution channels will be studied with qualitative methods (focus group discussions, individual interviews and key informants interviews) during the intervention phase (rainy season 2007).

Study site

The study programme will be carried out in Nouna Health District in north-western Burkina Faso. Nouna Health District has a population of around 250.000. The capital town is Nouna with some 25.000 population. The Nouna area is a dry orchard savannah, populated mainly by subsistence farmers of different ethnic groups living in small villages. Malaria is holoendemic but highly seasonal. Formal health services are limited to twenty-second village-based rural health centres each serving a catchment area of 6-10 villages, and to the district hospital in Nouna town. Until today, ITN are only available for young children enrolled into the D4 cohort study in the catchment area of the four health centres in the rural CRSN study area (population 35.000), and to a small proportion of households in Nouna town. Here a branded ITN version (PermaNet=Serena) is sold since two years in supermarkets and the main drug store.

Study work plan

During 2005, the following activities are planned:

- Further discussions of the Heidelberg and CRSN researchers with the director of the NMCP and PSI regarding the logistics of the study
- A local project management team (PMT) will be formed consisting of members from the CRSN, the DHT and PSI (where the DHT will be responsible for the ANC distribution and PSI for the social marketing distribution)
- The PMT will have a meeting with all heads of the 22 CSPS in Nouna explaining the goal of the study and discussing procedures. Thereafter all CSPS staff will sensitise the study population in all health centre catchment areas on the respective interventions (in case of the social marketing arm with support from PSI).
- The catchment areas of the 22 health centres will be randomised to either Intervention A or Intervention B
- The baseline sample will be on the respective interventions (in case of the social marketing arm with support from PSI)iscussion procedures. )0000000000000defined (village sample). Logistics for the first cross-sectional household survey will be discussed and prepared and the questionnaires designed.
- Design of the costing study

During 2006, the following activities are planned:

- The first cross-sectional survey will be carried out in February after training of respective field staff and preparation of all materials.
- The ITNs will be transported from Ouagadougou to Nouna town (March) and stored in the DHT warehouse until further transport (April/May) to the rural health centres (logistics to be defined).
- Distribution of ITNs free of charge to all pregnant women through the health centre-based ANC services of the intervention area A (starting in June 1^st^).
- Distribution of ITNs at a subsidised prize to the population through a shop-based distribution system in the frame of a PSI-supported social marketing approach (to be defined).
- Comprehensive data collection on process indicators (to be defined).
- Implementation of the costing study
- The first follow-up sample will be defined.
- Data entry, data cleaning.

During 2007, the following activities are planned:

- The second cross-sectional survey will be carried out in February after training of respective field staff and preparation of all materials.
- Knowledge, attitudes and practices (KAP) study in a random sample of the study population and in a provider sample (February/March/April).
- Continuation of distribution of ITNs free of charge to all pregnant women through the health centre-based ANC services of the intervention area A.
- Continuation of distribution of ITNs at a subsidised prize to the population through a shop-based distribution system in the frame of a PSI-supported social marketing approach.
- Continuation of data collection on process indicators and costing data.
- Validation study (on a sample of 100 households to be matched with the 2008 follow-up sample) on ITN use during pregnancy and infancy (August/September).
- The second follow-up sample will be defined.
- Data entry, data cleaning.

During 2008, the following activities are planned:

- The second cross-sectional survey will be carried out in February after training of respective field staff and preparation of all materials.
- The ITNs (100) for the entomological efficacy study sample will be collected during the second follow up survey (February). Laboratory tests to assess mosquito mortality will be conducted directly in Nouna by the two entomological assistants under the supervision of a professional entomologist to be contracted. Laboratory tests to assess insecticide content will be conducted by a specialised laboratory in Europe.
- Final data entry, data cleaning, data analysis, report writing.
- Presentation of study data to policy makers through a national workshop

Ethical considerations

The protocol has been reviewed and approved by the Ethics Committee of the University of Heidelberg and the local Ethics Committee in Nouna, Burkina Faso.

Community consent will be sought from the relevant local authorities of Nouna Health District prior to the start of the trial. The population will be informed about the project through the health workers of the peripheral health centres and through local radio stations. Informed consent will be sought before any interview. Findings of the study will be shared not only with the local and national health authorities, but also with the population.
